# Correlation Between Subjective Happiness and Pleasant Activities at Workplace in Nursing Staff for Older Individuals in Japan

**DOI:** 10.1007/s10597-019-00539-w

**Published:** 2019-12-28

**Authors:** Shinya Takeda, Shigeki Nakayama, Md. Sahab Uddin, Atsumi Hiramoto, Masahiko Inoue

**Affiliations:** 1 Department of Clinical Psychology, Tottori University Graduate School of Medical Sciences, Tottori, Japan; 2 National Institute of Technology, Yonago College, Tottori, Japan; 3 Department of Pharmacy, Southeast University, Dhaka, Bangladesh; 4 Pharmakon Neuroscience Research Network, Dhaka, Bangladesh; 5 Center for Research on Human Development and Clinical Psychology, Hyogo University of Teacher Education, Hyogo, Japan

**Keywords:** Nursing staff, Mental health, Subjective happiness, Pleasant activities, Workplace

## Abstract

The purpose of this study was to investigate the correlations between nursing staff’s mental health, number of years worked, night shifts performed, and pleasant activities at the workplace. One hundred forty-three subjects who had no missing data were analyzed. Questions consisted of basic attributes, subjective happiness scale (SHS), and pleasant activities conducted at the workplace. Denouements of SHS indicated a significant trend for the main effect, with more pleasant activities in the high SHS group than the low SHS group. The interaction was significant, with fewer pleasant activities in participants in the low SHS group who worked the night shift compared with those who worked the night shift regardless of work experience. The outcomes of this study suggest that it is essential to expand the repertoire of pleasant activities at the workplace to increase the subjective happiness of nursing staff for older individuals.

## Introduction

As the elderly population is expected to increase worldwide (United Nations 2017), securing personnel to provide care to elderly individuals with various diseases, such as dementia, has become a significant issue. Japan has become the first country to be regarded as a super-aging society. Nursing staff who offer care for elderly individuals play an important role. However, an increasing number of nursing staff in recent years have been taking breaks from work or leaving their jobs (Castle and Engberg 2006; Chan et al. 2013; Hirata and Harvath 2015). One issue considered to be a cause of this trend is mental health problems. Particularly noteworthy is the increasing number of patients with depression. Additionally, as the labor shortage at elderly care facilities is becoming more serious, the burden on remaining nursing staff increases when others take time off work or leave their jobs. There is a fear that this will affect the quality of nursing care services. Nursing staff members are susceptible to stress (Chan et al. 2000; Bonde 2008; Alenezi et al. 2018), and it has been shown that workplace stress increases the risk of personnel taking long-term leave due to depression (Inoue et al. 2010). Accordingly, maintaining and improving the mental health of nursing staff is vital to prevent the temporary or permanent loss of personnel due to depression and to increase the quality of nursing care services for residents.

Various strategies have previously been implemented to maintain and improve mental health among nursing staff. For example, one study targeting nursing staff reported that situational anxiety can be improved by utilizing cognitive restructuring and relaxation (Yung et al. 2004), while subjective happiness can be enhanced through mindfulness (Mackenzie et al. 2006; Goodman and Schorling 2012). Additionally, it has been shown that the stress reaction of nursing staff can be improved by an on-the-job training program to enhance knowledge about dementia and care methods (Takizawa et al. 2017). However, nursing care is extremely demanding, and irregular work hours, including night shifts, make it difficult for nursing staff to be active on their days off. As a result, access to conventional programs, including those promoting mental health through learning new skills, is limited. Additionally, nursing staff that has poor psychological health have little motivation to learn new skills to maintain and improve their mental health (Takeda and Ota 2014). Therefore, we need strategies to preserve and promote mental health, which can be easily implemented between busy work periods and without an additional burden upon the nursing staff.

Interestingly, according to the applied behavior analysis model, behavior arises because of interaction with one’s environment and does not only rely on individual effort (Skinner 1981). Instead, behavior modification can be elicited by manipulating a person’s environment. It has been reported that behavior that results in a pleasurable feeling is a protective factor against depression (Hunnicutt-Ferguson et al. 2012). Given workplace behavior, behavior that brings about a pleasurable feeling can be understood as implemented to make work more enjoyable. Therefore, in this study, pleasant activities were defined as behaviors being implemented to make the workplace more pleasant for the staff. Clarifying the correlation between pleasant activities at the workplace and mental health could offer realistic strategies for maintaining and improving the mental health of nursing staff.

Moreover, as nursing care is highly physically demanding and involves numerous stressful situations, many staff find it challenging to work for an extended period of time. Accordingly, if changes in the workplace include pleasant activities for long- and short-term nursing staff, working healthily could be implemented in stressful nursing care workplaces. Meanwhile, it has been indicated that the results are affected by whether the nursing staff work under stressful night shifts (Coffey et al. 1988). When an adequate number of personnel have not been secured for night shifts, nursing staff working night shifts are under substantial pressure. Therefore, it is expected that whether nursing staff work in night shifts will also affect pleasant activities in the workplace. Based on the aforementioned point, it appears that investigating correlations between the mental health of nursing staff and pleasant activities at the workplace, depending on the number of years worked, and whether night shifts were performed, could provide valuable information to support the maintenance and improvement of mental health of nursing staff. However, no previous studies have investigated such correlations.

Therefore, this study’s purpose was to investigate these two issues. Firstly, correlations between the nursing staff’s mental health, the number of years worked, night shifts performed, and pleasant activities at the workplace were investigated. In this study, subjective happiness was used as an index for mental health because low subjective happiness has been shown to increase the risk of suicide and is believed to be strongly related to mental health (Koivumaa-Honkanen et al. 2001). The subjective happiness of nursing staff is also correlated with lifestyle satisfaction (Gurková et al. 2011); thus, it appears that subjective happiness is an essential factor in enabling nursing staff to continue working in a healthy manner. The factors related to happiness at the workplace for nursing staff has previously been studied. High subjective happiness at workplace in nursing staff correlates with extraversion and agreeableness (Karl et al. 2007), high job satisfaction (Gurková et al. 2014), qualities of hardiness (Abdollahi et al. 2014), healthy lifestyle (Oates 2018), lower task requests (Hwang 2019), and prayer (Achour et al. 2019). Secondly, qualitative differences in pleasant activities at the workplace were investigated for elements that exhibited statistically significant differences.

## Methods

### Subjects

Nursing staff working at care facilities for older individuals in Tottori and Yamaguchi Prefectures (approximately 400 km apart) were targeted to eliminate regional bias. A questionnaire was distributed to 214 individuals who participated in a workshop for nursing staff from June to October 2017. Responses were received from 151 subjects who agreed to participate in the study (response rate: 70.6%). Of the 151 participants who provided consent, the responses of 143 participants with no missing data were analyzed (mean age: 43.4 ± 11.3 years; men: 40, women: 103).

### Questions

#### Basic Attributes

Participants were asked about age, sex, number of years of work experience as nursing staff, and whether they worked night shifts. Work experience was categorized as less than 1 year, 1 year to fewer than 5 years, 5 years to fewer than 10 years, and 10 years or longer. Night shift work was categorized as yes or no.

#### Subjective Happiness

The subjective happiness scale (SHS; Lyubomirsky and Lepper 1999) was used. This scale is designed to determine the characteristics of people who remain happy despite their circumstances (Lyubomirsky et al. 2001). The present study aimed to determine the behavioral characteristics of people who remained happy in stressful nursing care workplaces. Scores for the SHS ranged from 4 to 28 points, with a higher score indicating a higher level of subjective happiness.

#### Pleasant Activities in the Workplace

Participants were asked, “What action do you routinely take in order to make work enjoyable?” They provided open-ended responses regarding behavior (pleasant activities) that they implemented to enjoy their work. For statistical analysis, the number of items cited in open-ended responses was used as indices.

#### Ethical Considerations

Participants received an overview of the study, and written explanations that data gathered in this study would be analyzed so that individuals could not be identified, only those who consented would be examined, and no disadvantages would arise because of consenting or not consenting to participate in the study. The informed consent of the participants was then obtained.

### Statistical Analysis

The normality of the data was examined using the Shapiro–Wilk test. In the present study, analysis of variance (ANOVA) was used to investigate the main effect of each variable and the interaction between the nursing staff’s subjective happiness, work experience, and night shift work. Three-way ANOVA was performed using SHS, work experience, and night shift work as independent variables and the number of pleasant activities as a dependent variable. The three factors, as independent variables, were grouped as follows. Participants were divided into three groups based on the SHS score percentile (low SHS group, medium SHS group, and high SHS group), two groups based on less than 10 years of work experience or 10 years or more of work experience, and two groups based on whether or not they worked at night shift. On the other hand, qualitative data were classified using the KJ grouping method (Scupin 1997). The KJ method was used by a total of three individuals, including one university faculty member specializing in nursing staff stress management and two nursing staff with at least 10 years of experience.

## Results

### Correlations Between Pleasant Activities at the Workplace and Subjective Happiness, Work Experience, and Night Shift Work

The Shapiro–Wilk test confirmed that the data indicated normal distributions. There were 53 participants in the low SHS group, 55 in the medium SHS group, and 35 in the high SHS group. There were 70 participants in the < 10 years of work experience group and 73 in the ≥ 10 years of work experience group. Seventy-two participants worked night shifts and 71 who did not (Table 1). Results of SHS indicated a significant trend for the main effect (F [2,142] = 2.6, p < 0.10), with more pleasant activities in the high SHS group than in the low SHS group. The main effect of working night shifts was significant (F [1,142] = 9.4, p < 0.01), with fewer pleasant activities for participants who worked night shifts than for those who did not. The interaction was found to be significant (F [2,142] = 4.3, p < 0.05), with fewer pleasant activities for participants in the low SHS group who worked the night shift compared with those who worked the night shift regardless of work experience.

**Table 1:** Means and standard deviations of number of pleasant activities

	Subjective happiness			Work experience		Night shift work	
	Low group (n = 53)	Medium group (n = 55)	High group (n = 35)	Less than 10 years (n = 70)	10 years or more (n = 73)	Yes (n = 72)	No (n = 71)
Number of pleasant activities	1.7 (1.2)	1.9 (1.1)	2.3 (1.1)	1.8 (1.0)	2.0 (1.2)	1.6 (1.0)	2.2 (1.1)

Standard deviations are in parentheses

### Qualitative Investigation of Pleasant Activities by Subjective Happiness and Night Shift Work

Statistical analysis revealed differences in the number of pleasant activities depending on the degree of subjective happiness and night shift work. Therefore, participants were classified into four groups: high subjective happiness/night shift, high subjective happiness/no night shift, low subjective happiness/night shift, and low subjective happiness/no night shift. Qualitative differences were investigated in the pleasant activities being implemented by nursing staff depending on combinations of high or low subjective happiness and whether they worked the night shift (Table 2).

**Table 2:** Sample items in each category

Group	Category	Item
High subjective happiness/night shift	Greetings	Giving greetings with a loud voice
		Giving greetings with a smile
		Giving greetings first
	Mental diversions	Wearing one’s favorite shoes
		Listening to one’s favorite music during break time
		Scribbling on things before throwing them away
	Smiling	Smiling at people while interacting with them
		Making small jokes and laughing
	Reframing	Accepting everything positively and taking action
		Considering even negative things positively
		Considering problems positively
	Moving efficiently	Adjusting one’s schedule to make it possible to return home at a regular time
		Planning to ensure that work was not too difficult
		Following instructions and remaining calm while working
	Engaging in conversation	Speaking to residents
		Making light conversation with easygoing personnel
		Speaking to personnel and residents
	Finding good points in other people	Finding good points in personnel
		Making sure to praise peoples’ good points
		Finding things that residents were good at or enjoyed and making these a topic of conversation
	Engaging with people in accordance with their type	Being kind, avoiding negative words and actions
		Adjusting one’s approach depending on the person’s facial expressions
High subjective happiness/no night shift	Greetings	Giving greetings with a loud voice
		Giving greetings with a smile
		Giving greetings first
	Mental diversions	Watering flowers
		Relaxing
		Listening to favorite songs
	Smiling	Smiling at people while interacting with them
		Making small jokes and laughing
	Reframing	Considering whether there were good aspects to disliked traits of a person’s personality
		Considering methods of making things possible rather than reasons that things were impossible
		Focusing on fun aspects rather than unpleasant aspects of work
	Moving efficiently	Planning to ensure that each day’s work proceeded smoothly
		Keeping overtime work to within two hours per month
		Tidying up and making preparations to ensure that each day’s work proceeded smoothly
	Engaging in conversation	Enjoyably conversing with residents
		Engaging in communication
		Starting conversations
	Finding good points in other people	Conveying a person’s positive changes to them
		Praising a person’s good points
	Maintaining harmony with colleagues	Getting along with personnel
		Sharing with colleagues what they tried to do and what was fun
		Considering what one’s colleagues wanted
Low subjective happiness/night shift	Greetings	Giving greetings with a loud voice
		Giving greetings with a smile
		Giving greetings first
	Mental diversions	Thinking of fun things
		Changing one’s mood by going to one’s favorite place
		Moving one’s hands
	Moving efficiently	Arriving at one’s workplace 15 min early
		Considering the day’s work flow
	Maintaining harmony with Colleagues	Adjusting to fit in with others
		Dealing with all personnel in the same manner
	Establishing goals	Determining personal goals
		Determining goals each day
	Smiling	Smiling at people while interacting with them
		Making small jokes and laughing
	Finding good points in other people	Finding good points in personnel
		Making sure to praise peoples’ good points
	Engaging in conversation	Enjoyably conversing with residents
		Engaging in communication
Low subjective happiness/no night shift	Establishing goals	Determining goals each day
		Determining weekly goals
	Smiling	Smiling at people while interacting with them
		Making small jokes and laughing
	Greetings	Giving greetings with a loud voice
		Giving greetings with a smile
		Giving greetings first
	Engaging with people in Accordance with their type	Treating residents kindly
		Listening to all that residents have to say
		Gathering topics of conversation in accordance with others
	Considering the needs of personnel	Help personnel who are in trouble
		Aid personnel in their work duties
		Check in on colleagues who look unhappy
	Mental diversions	Making sure to take breaks
		Things to do in a notebook and noting when they are finished
		Not planning too much work
	Finding good points in other people	Finding one good thing about another person
		Making positive comments
	Engaging in conversation	Engaging in communication
		Starting conversations

Based on the results, eight categories were extracted for the high subjective happiness/night shift classification. These were: (1) “greetings,” (2) “mental diversions,” (3) “smiling,” (4) “reframing,” (5) “moving efficiently,” (6) “engaging in conversation,” (7) “finding good points in other people,” and (8) “engaging with people in accordance with their type.” Eight categories were also extracted for the high subjective happiness/no night shift classification. These were: (1) “greetings,” (2) “mental diversions,” (3) “smiling,” (4) “reframing,” (5) “moving efficiently,” (6) “engaging in conversation,” (7) “finding good points in other people,” and (8) “maintaining harmony with colleagues.” Eight categories were identified for the low subjective happiness/night shift classification. These were: (1) “greetings,” (2) “mental diversions,” (3) “moving efficiently,” (4) “maintaining harmony with colleagues,” (5) “establishing goals,” (6) “smiling,” (7) “finding good points in other people,” and (8) “engaging in conversation.” Finally, for the low subjective happiness/no night shift classification, nine categories were identified. These were: (1) “establishing goals,” (2) “smiling,” (3) “greetings,” (4) “engaging with people in accordance with their type,” (5) “considering the needs of personnel,” (6) “mental diversions,” (7) “moving efficiently,” (8) “finding good points in other people,” and (9) “engaging in conversation.”

## Discussion

### Differences in Pleasant Activities at Workplace Depending on Nursing Staff’s Subjective Happiness, Work Experience, and Night Shift

The first aim of this study was to investigate the correlations between behavior at the workplace and nursing staff’s mental health, work experience, and night shift. The results suggest that higher subjective happiness in nursing staff is associated with more pleasant activities at the workplace. SHS, which was used in this study, is a scale to determine the characteristics of people who can remain happy despite various circumstances (Lyubomirsky et al. 2001). Our results indicated that more pleasant activities at the workplace were seen in study participants with higher subjective happiness despite engaging in stressful nursing care. This suggested that pleasant activities at the workplace affect the subjective happiness of the nursing staff. The subjective happiness of nursing staff has been correlated with lifestyle satisfaction (Lin et al. 2010). The implementation of pleasant activities in the workplace may increase workplace satisfaction. Therefore, pleasant activities at the workplace appear to be a factor for increasing subjective satisfaction.

The results of the study showed that nursing staff who work in night shifts implement fewer pleasant activities at the workplace than the nursing staff who do not work during night shifts. Moreover, this trend was more marked in nursing staff with low subjective happiness. Nursing staff who work in night shifts may experience more stress than those who do not (Coffey et al. 1988). Because there is a definite shortage of nursing staff who can work in night shifts, it appears that such nursing staff does not have enough flexibility to implement pleasant activities. Therefore, an issue for future investigation is pleasant activities that can be implemented by nursing staff who work in night shifts to increase their subjective happiness.

Meanwhile, no significant correlation was observed between work experience, which was an independent variable, and pleasant activities at the workplace. Accordingly, our results did not demonstrate that having pleasant activities at the workplace is a factor supporting the long-term retention of the nursing staff. Many nursing staff members leave their jobs within a short period, which results in a labor shortage in the nursing care industry, thereby lowering the quality of nursing care services offered to residents. Long-term retention of nursing staff can influence the ability to secure human resources in the nursing care industry, making it an essential element in improving the quality of nursing care services. Factors supporting the long-term retention of nursing staff are an area that requires further investigation.

### Differences in Pleasant Activities at the Workplace Depending on Subjective Happiness and Night Shift Work

The second aim of this study was to investigate the qualitative differences in pleasant activities in the workplace by analyzing factors that exhibited significant differences. The factors for which significant differences were noted were subjective happiness and night shift work. Therefore, we investigated how these differences affected qualitative differences in pleasant activities.

A pleasant activity category shared by groups with high subjective happiness regardless of night shift work was “reframing.” Activities classified as reframing refer to attempts to change one’s viewpoint and look at things from a different perspective. Negative perceptions are known to promote depressive symptoms and inhibit active behavior (Iacoviello et al. 2006). Simultaneously, the cognitive model proposed by Beck indicates that mood improvement can be achieved by changing negative perceptions into realistic perceptions (Beck 1963). Thus, it has been noted that cognitive flexibility can improve mental health (Hayes 2004). Reframing involves cognitive modification in the sense of reconsidering things from a different viewpoint. It requires that one’s cognitive flexibility be trained. Cognitive flexibility is related to resilience, which is linked to subjective happiness (Arrogante and Pérez-García 1963). Accordingly, engaging in reframing the workplace may constitute behavior that increases subjective happiness among nursing staff, regardless of night shift work.

A pleasant activity category shared by groups with low subjective happiness regardless of night shift work was “establishing goals.” Goal-setting is generally believed to increase motivation, and achieving goals can provide opportunities to feel happy. However, the fact that there were many attempts at “establishing goals” among individuals with low subjective happiness in this study suggested that depending on the method used, setting goals may lower subjective happiness. For example, establishing goals that are too high makes it difficult to achieve them, thereby making it impossible for an individual to experience a sense of achievement. Many people in Japan are thought to have the cognitive characteristics of pursuing perfection and dichotomous thinking. Setting goals that are too high or pursuing perfection may quickly reduce subjective happiness because it may result in a negative self-perception when unrealistic goals cannot be achieved. Accordingly, future investigations need to be conducted regarding the qualitative characteristics of goal-setting to clarify activities that produce low subjective happiness.

“Maintaining harmony with colleagues,” which was identified as a category, was shared by the high subjective happiness group that did not work in night shifts and low subjective happiness group that did work in night shifts. This result suggests that making efforts to maintain good relationships among personnel may increase subjective happiness when there is no night shift work, but may decrease subjective happiness when there is night shift work. Moreover, the six categories of “greetings,” “mental diversions,” “smiling,” “moving efficiently,” “engaging in a conversation,” and “finding good points in other people” were shared among all the groups regardless of night shift work or the extent of subjective happiness. It has been shown that implementing healthy behavior and receiving positive reinforcement can reduce depression in individuals (Shiota et al. 2017). Positive reinforcement provides desirable stimulation for individuals. From this, it can be inferred that even for instances of the same behavior, the results that occur immediately afterward can result in different effects on subjective happiness. In this study, subjective happiness was found to be both high and low for the same behavioral categories. Accordingly, the correlation between subjective happiness and environmental reinforcement after certain pleasant activities needs to be investigated in the future.

## Limitations of the Study

The findings of this study must be understood with methodological limitations. The sample size limits suggestion to generalizability. In addition, the present study cannot mention the causality between subjective happiness and pleasant activities at the workplace. Therefore, further research should examine whether pleasant activities at the workplace can enhance the mental health of nursing staff for older individuals, and to consider strategies that allow nursing staff with low mental health to efficiently perform pleasant activities at the workplace.

## Conclusions

This study suggested that it is crucial to expand the repertoire of pleasant activities at the workplace to increase the subjective happiness of nursing staff for older individuals. Moreover, it appears that many nursing staff members with high subjective happiness are implementing reframing. Reframing, which involves reconsidering things from a different viewpoint, seems to be a powerful pleasant activity that increases the subjective happiness of nursing staff for older individuals. Therefore, strategies to enable the nursing staff to quickly implement reframing need to be investigated.
